# Metabolomic and metagenomic analysis of Intestinal Microbiota in the dysbiosis of type 2 diabetes mellitus and Alzheimer disease

**DOI:** 10.1002/alz70856_097688

**Published:** 2025-12-24

**Authors:** Ángel Radamés Rábago Monzón, Juan Fidel Osuna Ramos, Yair Cruz Narvaez, Maribel Hernández Rosales

**Affiliations:** ^1^ Universidad Autónoma de Sinaloa, Culiacán, SI, Mexico; ^2^ Instituto Politécnico Nacional, Cdmx, EM, Mexico; ^3^ CINVESTAV, Irapuato, GJ, Mexico

## Abstract

**Background:**

T2DM and AD are major public health concerns characterized by metabolic and cognitive impairments, respectively, with growing evidence suggesting that gut microbiota alterations contribute to their pathogenesis. Metagenomic and metabolomic analyses provide valuable insights into the microbiota's role in glucose regulation, inflammation, and dementia risk, offering potential for early diagnosis and targeted interventions. Understanding the interplay between gut microbiota and metabolic pathways could lead to novel therapeutic strategies to improve patient outcomes.

**Method:**

A descriptive study with a quantitative approach, cross‐sectional observational comparative, of relational scope will be conducted. The study population will be segmented into four groups and two subgroups: Control (CTRL) (*n* = 30), Type 2 Diabetes Mellitus (T2DM) (*n* = 30), Alzheimer's Disease (AD) without T2DM (*n* = 30), and AD with T2DM (*n* = 30). Subgroups include Control (Young adults) (*n* = 30) and T2DM (Young adults) (*n* = 30). All groups will undergo characterization, which includes blood chemistry, and clinical, mental, nutritional, and anthropometric evaluations. We obtained urine and stool samples for DNA extraction and library preparation. We used Magnetic Resonance Mass Spectrometry (MRMS) for metabolomic analysis, which uses eluents to detect metabolites. We will apply MetaHit bioinformatics tools to assess sample diversity and perform metabolomic analysis in RStudio.

**Result:**

The study revealed distinct patterns of intestinal dysbiosis and metabolic changes in patients with T2DM and AD, categorized by age. A comprehensive taxonomic and functional representation of the gut microbiome highlighted condition‐specific differences. Significant correlations were found between microbiological, metabolomic, and clinical biomarkers, particularly those related to cognitive decline. Key metabolic pathways and molecular processes underlying dysbiosis were identified. Fecal metabolite analysis uncovered distinctive compounds such as (+/‐)‐Ethylketocyclazocine, (‐)‐Quebrachamine, and (‐)‐jasmonoyl‐L‐isoleucine, while urinary metabolites like (Phenylthio) acetic acid and 2,3‐Diketo‐L‐gulonate showed disease‐associated variations. These findings support the development of personalized interventions to mitigate cognitive decline through microbiota and metabolomic profile modifications.

**Conclusion:**

The study identifies distinct gut microbiota and metabolic patterns linked to cognitive decline in T2DM and AD, offering insights into disease mechanisms and supporting the development of personalized therapeutic strategies to improve patient outcomes.

